# High-Throughput Sequencing for the Detection of Viruses in Grapevine: Performance Analysis and Best Practices

**DOI:** 10.3390/v16121957

**Published:** 2024-12-20

**Authors:** Kristian A. Stevens, Maher Al Rwahnih

**Affiliations:** 1 Department of Computer Science, University of California-Davis, Davis, CA 95616, USA; 2 Department of Evolution and Ecology, University of California-Davis, Davis, CA 95616, USA; 3 Foundation Plant Services, University of California-Davis, Davis, CA 95616, USA; malrwahnih@ucdavis.edu; 4 Department of Plant Protection, School of Agriculture, The University of Jordan, Amman 11942, Jordan; 5 Department of Plant Pathology, University of California-Davis, Davis, CA 95616, USA

**Keywords:** plant viruses, bioinformatics, NGS, grapevine, diagnostics

## Abstract

Among the cultivated crop species, the economically and culturally important grapevine plays host to the greatest number of distinctly characterized viruses. A critical component of the management and containment of these viral diseases in grapevine is both the identification of infected vines and the characterization of new pathogens. Next-generation high-throughput sequencing technologies, i.e., HTS technologies, have been widely adopted for their ability to quickly, broadly and directly characterize molecular sequences associated with potential pathogens. We empirically analyze the performance of HTS as a diagnostic tool in a phytosanitary context and make recommendations on its deployment for detecting known and novel viruses in grapevine. Three popular and widely used modalities for analyzing HTS data are characterized and compared using the standard diagnostic performance criteria of sensitivity (the true positive rate), specificity (the true negative rate) and analytical sensitivity (dilution series).

## 1. Introduction

Among the cultivated crop species, grapevine plays host to the greatest number of distinct viruses [1]. Over 101 plant viruses from diverse families have been characterized in this economically and culturally important crop [2,3]. The majority of viruses isolated from grapevine have *Vitis* sp. as the only known host [2]. More importantly, most of the viruses isolated from grapevine are associated with damaging symptoms, about half of which are widespread [2].

A critical component of the management of these viral diseases is the identification and subsequent removal of infected vines. This happens at the vineyard level, in quarantine programs and at the production level. In particular, the establishment of virus-tested foundation vine-stock as a resource for propagation is an essential component in the production of quality planting materials and for the planting of healthy vineyards. Thus, clean plant and quarantine facilities like Foundation Plant Services (FPS) are important consumers of virus detection technology. Located at the University of California, Davis, FPS is a USDA-APHIS-permitted grapevine importation center and maintains a virus-tested collection of grapevine totaling more than 2400 selections. Each selection is rigorously tested for pathogens before release from quarantine and inclusion in the collection.

### 1.1. Identification of Grapevine Virus Infections

The conventional serological and molecular methods for detecting viral infections are enzyme-linked immunosorbent assay (ELISA) and Polymerase Chain Reaction (PCR), respectively. These methods are widely considered sensitive, but they require prior knowledge of the virus to develop. Because developing an ELISA is a time-consuming and expensive process, PCR is the more favored of the two but not without its own drawbacks. PCR primers are highly sequence-specific; thus, developing a sensitive and specific PCR assay requires detailed knowledge of both the genome and nucleotide diversity of the target virus. The high sequence specificity of PCR primers makes designing assays for diverse virus species a unique challenge [4]. It can also lead to false-negative test results in the presence of unanticipated genetic diversity [5,6].

Biological indexing has been the standard in quarantine and regulatory programs for decades; it has the ability to detect viral infections independent of specific knowledge of the infecting virus, as is the case for novel viruses. This method involves the replicated grafting of subjects onto indicator plants followed by years of expert monitoring for virus symptoms. Biological indexing is expensive, time consuming, and subject to false-negative results [7].

In this context, next-generation high-throughput sequencing technologies, i.e., HTS technologies, have been widely adopted for their ability to quickly, broadly, and directly characterize molecular sequences associated with the potential pathogen. The primary advantage of HTS over the aforementioned methods is that deep sequencing provides a detailed view of the infection status of the plant. Because the nucleotide sequence data are available, taxonomic characterization can be accomplished in silico using approaches that are much more robust to nucleotide divergence. Novel viruses can be identified and characterized using translated comparisons that infer amino acid similarities over very long evolutionary distances.

### 1.2. This Study

In this study, we empirically analyze the performance of high-throughput sequencing as a diagnostic tool and make recommendations on its deployment for detecting known and novel viruses in grapevine. Previous quality assessments and validation of sampling, timing, internal controls and sample templates were completed by [8]. This study provides a deeper look at the bioinformatics on which the diagnostics rely. Many of our recommendations have been featured in the HTS bioinformatics workflow at FPS, which is the ensemble method presented at the end of this paper.

Three widely used modalities for analyzing HTS data are characterized and compared. First, the mapping of reads to a reference database, which requires a priori knowledge of the virus species genome. Second, to facilitate the detection of novel viruses, we employ a de novo assembly approach. Finally, we looked at a more recent metagenomic method that relied on a database of taxonomically classified short k-length substrings (i.e., k-mers) to taxonomically classify sequenced reads [9]. We evaluated these approaches using the standard diagnostic performance criteria of sensitivity (the true positive rate) and specificity (the true negative rate) [10,11].

## 2. Material and Methods

### 2.1. Grapevine Panel Selection

Our validation panel consists of 19 grapevine plants infected by a broad range of common DNA and RNA viruses and viroids (Table 1). Eighteen virus-infected plants were selected for this study because of their consistent use as positive controls for diagnostic testing at FPS [12]. They are also proximally maintained in the Davis Virus Collection [13]. The panel also includes one virus-free sample that is only infected with Hop stunt viroid (cv. ‘Ganzin’) maintained as a rootstock in a foundation vineyard at FPS. All vines were tested by HTS and real-time reverse transcription quantitative PCR (RT-qPCR) or qPCR, as described previously [5,14]. The phytosanitary status of these plants is given in Supplementary Table S1.

### 2.2. TNA Extraction

Petioles and dormant canes were collected from the 19 vines in late May and October (spring and fall), respectively. Petioles or cane scrapings (665 mg/sample) were processed and spiked with 35 mg of leaf tissue from *Phaseolus vulgaris* endornavirus 1- and 2-infected common bean to act as a positive control for extraction and library preparation (see [8]). The spiked tissue was subsequently homogenized in 10 mL lysis buffer (4 M guanidine isothiocyanate; 0.2 M sodium acetate, pH 5.0; 2 mM EDTA; 2.5% (*w*/*v*) PVP-40) using a Homex grinder (Bioreba, Reinach, Switzerland) followed by TNA extraction with the MagMax Plant RNA Isolation kit (ThermoFisher Scientific, Waltham, MA, USA), excluding DNase treatment. TNA was quantified with the Qubit (ThermoFisher Scientific, Waltham, MA, USA).

### 2.3. Illumina Library Preparation and Sequencing

For individual samples, a total of 700 ng per 10 µL of extracted nucleic acids was subjected to rRNA depletion (only for TNA-based input) and cDNA library construction. Later, cDNA libraries were end-repaired, adapter-ligated by unique dual indexes, PCR-enriched, and used in two separate HTS runs. Finally, the amplicons were sequenced in an Illumina NextSeq 500 platform using a single-end 75 bp format. To reduce carryover from the previous run, three washes were performed prior to sample loading.

### 2.4. In Silico Dilution Series

To increase the tested range of virus titers and investigate the limits of detection for these methods, we created an extensive in silico dilution series. The reads from each fully sequenced sample were randomly subsampled to create in silico subsampled replicates. Sampling was performed without replacement until replicate dilution occurred or the sequence was exhausted. For each sample, we created the following 32 subsampled replicates (K = thousand reads): 150 K, 250 K, 500 K, 1000 K, 1500 K, 2000 K, 2500 K, 3000 K, 3500 K, 4000 K, 4500 K, 5000 K, 6000 K, 7000 K, 8000 K, 9000 K, 10,000 K, 11,000 K, 12,000 K, 13,000 K, 14,000 K, 15,000 K, 16,000 K, 17,000 K, 18,000 K, 19,000 K, 20,000 K, 21,000 K, 22,000 K, 23,000 K, 24,000 K, 25,000 K.

### 2.5. Sequence Processing

All Illumina reads were demultiplexed using bcl2fastq v2.20.0.422. The tolerance for barcode mismatches was set to 0 to reduce the possibility of crosstalk (or misidentified barcodes) between adjacent clusters on the flow cell. Adapter trimming is also performed by bcl2fastq during demultiplexing. Where noted, we also evaluated the added utility of using trim galore (v 0.6.7) as an additional adapter and quality trimming step.

Where noted, we performed a host genome screen by mapping sequenced reads against a target database consisting of the grapevine (*Vitis vinifera*) reference genome (GCF_000003745.3), mitochondrion (NC_012119) and chloroplast (NC_007957). Mapping was conducted using bowtie2 [15], and all exact matches were filtered.

### 2.6. De Novo Assembly

Three commonly used de novo assemblers were evaluated for this project, namely megahit (v1.2.9), developed at the Beijing Genome Institute [16]; trinity (v2.13.2), developed at the Broad Institute and the Hebrew University of Jerusalem [17]; and spades (v3.15.4), also known as the Saint Petersburg genome assembler [18]. In all cases, de novo assemblers were run with default values for a single-read (unpaired) Illumina sequencing protocol. Assemblies were annotated using NCBI blastn, blastx, and DIAMOND blastx [19,20].

### 2.7. Read Mapping

Illumina reads were mapped to virus and viroid sequence databases using bowtie2 (v2.4.2) software [15]. Bowtie was run in two variations: with default parameters for unpaired reads and also in very sensitive local mode (bowtie2-very-sensitive-local), which allows for more divergent partial alignments using a local alignment algorithm with a scoring methodology that reduces the penalty for mismatches and gaps. Where noted, the pathoscope2 software package (v2.0.6) [21] was employed to reassign multi-mapped reads using the very sensitive local bowtie2 output, as described by the Pathoscope 2 method [21], and implemented for detecting plant viruses in [22].

### 2.8. Taxonomic Read Classification Using k-Mers

We used the program kraken2 [9] to perform read-based taxonomic classification. Reads were taxonomically classified using a database of taxonomically classified k-mers constructed using a kraken2-build from the GenBank non-redundant nucleotide database. Classifying reads using kraken2 is the most memory intensive method employed in this study due to the size of the database, and kraken2 can only be run on a computer with at least 1 Tb of ram installed. After reads were classified, read counts for each taxon were compiled by adapting the method provided in the KrakenTools software suite v1.2 [23] to sum reads over all taxon ids associated with a virus (Supplementary Table S1). A sample was considered a positive for a virus or viroid if it met the minimum read count threshold for the associated taxon (Supplementary Table S1); otherwise, it was considered negative.

## 3. Results

### 3.1. Samples and Sequencing

For this study, we relied on a validation panel consisting of 18 virus-infected grapevine samples and one additional grapevine sample free of virus infection but infected with Hop stunt viroid. The 19 selected samples represented a broad range of common DNA and RNA virus and viroid infections (Table 1; Supplementary Table S1) which have been confirmed by PCR. Two independent tissue and timepoint replicates were taken from each grapevine: spring petioles and fall canes (Table 2).

Our 38 replicates were sequenced to a minimum target depth of 20 million reads using a 75 bp single-end (one read per molecule) Illumina protocol. This was performed across two flow cells, resulting in 903 million reads total, in line with the per flow cell yield of 450 million reads we typically see at FPS for this protocol. For these samples, we obtained 24 million reads on average, with all but two of the 38 replicates exceeding 20 million reads.

For a robust comparison of methods, and to ascertain limits of detection, each independent sample was subsampled at 32 different dilution levels (see materials). This resulted in 1216 in silico dilution replicates, with the goal of having a similar sequencing depth for each replicate in a two orders of magnitude dilution series (Table 2). After this step, the total number of reads analyzed increased from 903 million to 12.4 billion. The in silico replicates were then processed through a second round of adapter and quality trimming—the first round having already occurred during the demultiplexing step. Consistent with the high-quality adapter-free dataset that emerged from Illumina’s bcl2fastq software, few data were lost during this phase. We observed a 0.02% reduction in the number of reads and a 0.7% reduction in the total number of bases in the dataset. Subsequent to this, an additional dataset was created with host-screened reads. Our libraries are constructed from total RNA and are expected to contain a substantial amount of RNA originating from the genome of the host, even after employing a hybridization-based ribo-depletion step during library construction. In this dataset, a majority of the data (67.7% of reads and bases) was identified as similar to, and thus likely transcribed from, the host nuclear and organelle genomes.

### 3.2. Read Mapping for Virus Detection

For read mapping, we evaluate three target sequence databases of increasing size and generality. The smallest and most specific is a database of reference genomes of grapevine viruses (Ref-GV) which contains only a single reference genome for each grapevine virus and viroid in our study (Supplementary Table S2). These sequences primarily come from the NCBI RefSeq database or GenBank (when a RefSeq entry does not exist). All segments of the virus are represented in this database, but only one isolate or variant is chosen for each virus—therefore, genetic diversity is not captured. To capture viral genetic diversity in the database, we used a database consisting of all sequences for each grapevine virus and viroid deposited in GenBank, as identified by their taxonomic ID (NT-GV). Finally, for the broadest scope, we used the entire viral division of GenBank (NT-Viral). We were unable to evaluate all of GenBank in this study due to the excessive computational cost.

For each of the given databases, we evaluated three mapping approaches. The widely cited bowtie2 was examined as a representative of a short-reads mapping approach. We also evaluated the pathoscope2 software package, which uses a Bayesian approach to reassign multi-mapped reads to the most likely (minimal) taxonomic distribution. Because the pathoscope algorithm uses bowtie’s very sensitive local output (bowtie2-vsl), we evaluated that method separately so we could separate the utility of the more sensitive mapping algorithm from taxon assignment.

Finally, we evaluated the utility of host screenings in this context by performing mapping with and without a host screening. For each approach, diagnostic statistics were calculated using a minimum read count of 20 and a minimum coverage of 200 bp, chosen as a one-size-fits-all cutoff averaged over all dilution samples (Supplemental Figure S2). The results are summarized in Table 3.

*Mapping Algorithm.* For all but one combination of host screening and database, bowtie2-vsl performed optimally or co-optimally in terms of sensitivity (TPR); the only exception was NT-GV with no target host screening, where bowtie2 performed slightly better (delta TPR 0.18%). For all combinations of host screenings and databases, pathoscope2 performed optimally in terms of specificity (TNR). The pathoscope2 algorithm worked best on the database of grapevine virus reference genomes, similar to how it is deployed in [18]. In these two cases (with and without host screening), the reassignment of reads maintained sensitivity and increased specificity.

*Database Choice.* For the bowtie algorithms, the single-reference database performed less optimally in terms of sensitivity (TNR). We observed an increase in sensitivity and a decrease in specificity when moving to NT-GV, which incorporates more genetic diversity. Moving to NT-Viral, there is a slight increase in sensitivity (TPR) and specificity (TPR). The pathoscope algorithm performed optimally in terms of sensitivity and specificity on the single-reference database (Ref-GV), suggesting that the larger, less curated databases may confound the Bayesian assignment algorithm.

*Host filtering*. For all mapping algorithms, the unscreened reads performed the best in terms of sensitivity for the larger databases. If the goal is maximum sensitivity, mapping unscreened reads against a database incorporating genetic diversity (NT-GV and NT-Viral) performed best. On the flip side, the host-screened data mapped against the single-reference database (Ref-GV) using pathoscope2 had the best specificity.

*Sequencing depth.* The in silico dilution series was employed to determine a recommended sequencing depth. Using that data, we performed a limit of detection style analysis by plotting sensitivity (TPR) against sequencing depth (Supplemental Figure S1). The result for pathoscope against Ref-GV is included in the ensemble analysis (Section 3.6). The curves have an asymptotic quality, and, at 15 M, an obvious flattening of the curve occurs, a notable feature of all detection methods. We observed diminishing returns by 15 M reads, with sensitivity improving at only 1% per 5 million reads (Supplemental Figure S1).

### 3.3. De Novo Assembly for Virus Detection

**Figure 1:**
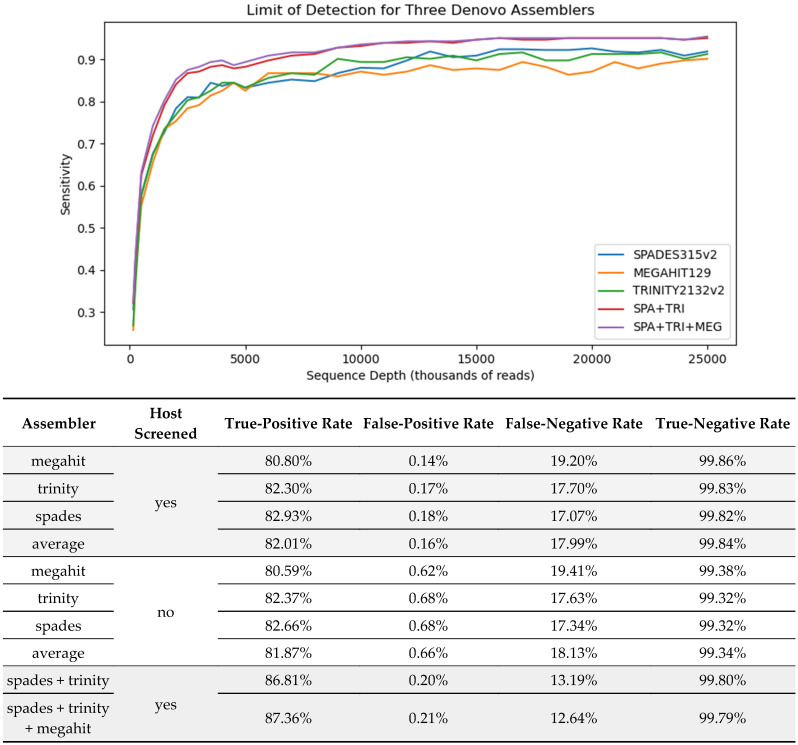
(**top**) Limit of detection style plot of sensitivity (TPR) over different in silico dilution levels. (**bottom**) Summary of diagnostic statistics for three popular de novo assemblers and two ensembles over all 1216 in silico replicates. Both screened and unscreened reads were considered for the individual assemblers.

We also looked at methods for de novo assembly of the overlapping reads from a metagenome and subsequent annotation of the resulting consensus sequences. The assembled genome consists of fewer but longer consensus sequences (contigs) that should allow for longer, more divergent homologies to be ascertained and for more computationally intensive annotation methods. Figure 1 shows the averaged diagnostic statistics and a limit of detection analysis for our de novo assemblers.

The results presented in the Figure 1 table suggest that, if one wanted to choose a single assembler for optimal sensitivity, it would be spades, which has the highest average TPR for the three assemblers evaluated over all 1216 in silico dilutions. The averaged results also support the idea that host screening may lead to higher sensitivity and specificity in a de novo assembly context. This is true for all three assemblers individually and averaged. A secondary benefit of host screening, for trinity in particular, is that it made the assembly much more tractable—as noted by a substantial decrease in runtime.

The limit of detection analysis shows the performance of each assembler with a host screening implemented as the number of reads sequenced is reduced. In regard to averaged overall sampling depths, the spades assembler performs the best in terms of sensitivity; however, it did not always maintain that rank for all sampling depths. For example, megahit performed best at the sampling depth of 6 million. The heuristic nature of assemblers suggests that trying more than one assembler on a dataset could be used to improve results. Indeed, we combined the results of the best two and three assemblers, such that a positive for one is a positive for the combination, to quantify this improvement. The shape of the curves in the LOD analysis also suggests diminishing returns in regard to sensitivity as the sequencing depth increases past 15 m reads, at which point the curves flatten out.

### 3.4. Comparing Annotation Methods

We evaluated tractable variants of BLAST [19] to annotate the contigs obtained from de novo assembly. In all cases, we used the appropriate GenBank non-redundant nucleotide (nt) and protein (nr) databases to annotate our sequences over a wide taxonomic range. This analysis was confined to the spades assembler after being identified as the single best option.

For annotating known viruses and viroids, NCBI blastn was employed. Overall, in silico subsamples of 15 million reads or more, the average sensitivity (TPR) was 92.18%. It was only slightly easier to detect viruses than viroids (delta TPR < 1%). This is probably due to their short lengths and the circular nature of viroid genomes.

For detecting novel viruses, we evaluated two tractable methods for translated searches. Both methods performed equally well in this study. In all the 1216 in silico replicates, the algorithms only differed in two annotations, both from in silico replicates with fewer than 15 million reads. Surprisingly, for replicates with 15 million or more reads, the results were identical (Table 4).

For detecting known viruses, blastn (TPR = 91.98%) is only slightly more sensitive than blastx (TPR = 91.92%). We expected larger differences due to the different alphabet sizes and scoring methods. We expect that these differences would become more pronounced for novel viruses as the level of divergence becomes higher and blastn is no longer effective. Not surprisingly, blastx is not suited for detecting viroids, so this comparison was only undertaken for viruses.

### 3.5. k-mer Methods for Virus Detection

**Figure 2:**
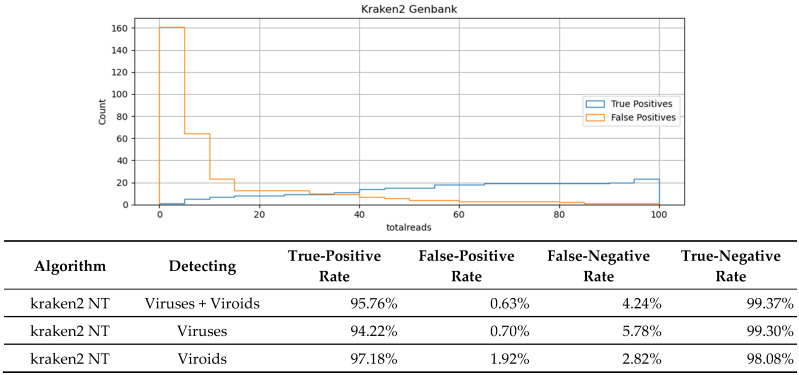
(**Top**) Histogram showing the number of taxonomically assigned reads associated with both true- and false-positive results in the in silico dilution data. (**Bottom**) Summary diagnostic statistics for the kraken2 method averaged over all in silico replicates sampled at a depth of at least 15 million reads.

We used the program kraken2 to perform virus detection using taxonomically classified reads using a database of taxonomically classified k-mers constructed from the GenBank non-redundant nucleotide database. A virus positive was determined if a specific read threshold is reached. The histogram in Figure 2 plots the number of taxonomically assigned reads associated with both true- and false-positive results in the in silico dilution data. From that plot, we assumed that 35 reads, the place where the TPR and FPR rates are roughly equal, would offer a good balance of sensitivity and specificity. The summary diagnostic statistics are presented in the Figure 2 table. The kraken2 algorithm performed nearly as well in terms of overall sensitivity as the best read mapping technique but with higher specificity, implying that it may actually perform better if the threshold was relaxed. The kraken2 algorithm performs better than de novo + blastn in terms of sensitivity at the expense of specificity.

### 3.6. Ensemble Methods

**Figure 3:**
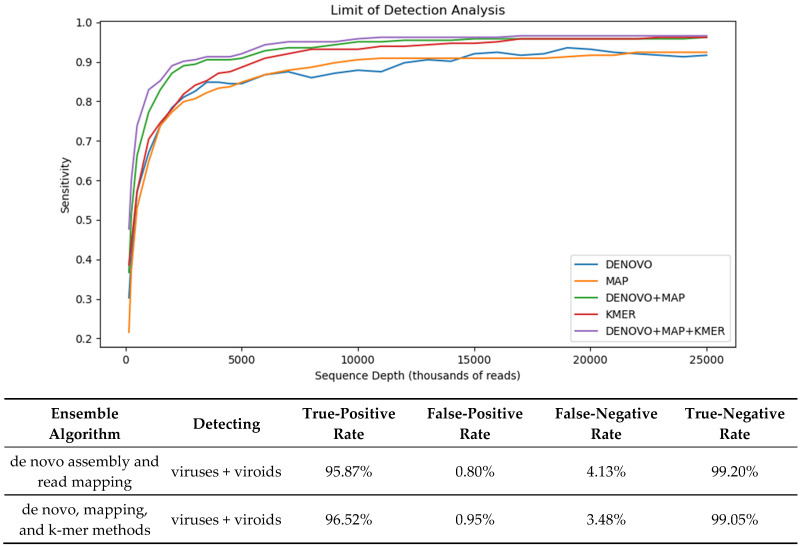
Ensemble methods. (**Top**) Limit of detection style plot of sensitivity (TPR) of the individual methods and the two ensemble methods over the different in silico dilution levels. (**Bottom**) Summary of diagnostic statistics for two ensemble methods averaged over all in silico replicates sampled at a depth of at least 15 million reads.

We evaluated the diagnostic performance of two ensemble methods in the context of a limit of detection analysis (Figure 3). A de novo assembly (spades + blastn + blastx) and read mapping (pathoscope2 + Ref-GV) method were combined logically by considering a sample positive for a viral agent if either method produced a positive result and negative only if both methods produced a negative result. This logic implies that the sensitivity can not decrease, since the ensemble set of positives is the union of the individual methods. Indeed, this is reflected in the results where the ensemble method is more sensitive (TPR = 95.9%) than both individual component methods (TPR = 92.18%) for de novo and (TPR = 92.25%) for read mapping. The price we pay for this increase in sensitivity is a decrease in specificity (TNR = 99.2%), as the ensemble set of false positives is the union of the false positives from the individual methods.

We extended our ensemble analysis to evaluate the ensemble of three methods: de novo, read mapping, and the kraken2 k-mer method. For this ensemble, a sample was considered a positive if any of the three methods produced a positive. By adding a k-mer method, sensitivity increased by a further 0.7% to an ensemble TPR of 96.52%, with specificity modestly decreasing by 0.2% to an ensemble TNR of 99.05%.

The limit of detection analysis illustrates that the ensemble methods particularly outperform the individual methods with dilutions of 5 million reads or less, which is notably less than a single order of magnitude from our target depth. We speculate the sensitivity gained by using an ensemble method to be even higher for low-titer infections.

## 4. Conclusions

In this paper, we compared the performance of multiple methods for ascertaining virus positives in grapevine using high-throughput sequencing (HTS). HTS promises to be an improved diagnostic technique for screening plant material and limiting the spread of material containing harmful viruses. It has been reported that HTS-based detection methods can perform at least as well or better than conventional biological and molecular methods under specific circumstances.

Previous studies have investigated different aspects of HTS performance criteria. Extraction protocols and sequencing platforms were investigated in [24,25]. Different nucleic acid templates were investigated in [25,26,27]. Bioinformatics pipelines have been investigated [25,26,27]. In this study, we evaluate the performance of different bioinformatics approaches for detecting both known and novel viruses in a diagnostic setting. We also evaluate ensemble approaches that can accomplish both. Finally, we make recommendations based on the findings in this study as well as the experience gleaned from processing thousands of individual samples [7].

### 4.1. Read Mapping Approaches

We believed that read mapping would always be a more analytically sensitive approach to detecting known viruses than de novo assembly. Theoretically, fewer reads are required to identify a positive compared to approaches that rely on de novo assembly to first construct a consensus sequence from multiple overlapping reads before classification can occur. Indeed, we have many anecdotes of PCR positives that are confirmed by a very small number of mapped HTS reads. When we employed cutoffs of 20 reads and 200 bp coverage, the read mapping method was not more sensitive or analytically sensitive than de novo, at least for known viruses. To obtain higher analytical sensitivity than de novo, we suggest lowering the minimum number of reads.

Our results showed that selection of the target database for read mapping is important. A single reference genome is not able to incorporate the high genetic diversity found in virus species. Target databases incorporating more than one reference genome per species outperformed target databases incorporating just a single reference in terms of sensitivity. We also showed that specificity can be increased by using the Pathoscope2 read-assignment technique. Pathoscope2 works by reassigning multi-mapped reads to a minimal number of virus species. This helps to reduce the number of false positives. The results suggest a tradeoff between diagnostic sensitivity (robustness to genetic variation) and analytical sensitivity (limit of detection). The high-quality single-reference database (Ref-GV) offers higher analytical sensitivity than the larger uncurated (NT-Viral) because the read cutoff can be lowered to achieve the same level of specificity.

### 4.2. De Novo Assembly Approaches

De novo assembly is important for identifying novel viruses. FPS researchers have discovered or supported the discovery of 35 novel viruses by employing de novo assembly followed by translated BLAST annotation [7]. However, de novo assemblers are very much heuristic methods which may be optimized for different scenarios. Our assessment of three de novo assemblers (for rnaSeq and metagenomic datasets) showed that choice of de novo assembler is an important consideration in terms of determining sensitivity. Spades and trinity performed clearly better than megahit at low coverage. Indeed, because assemblers may be optimized to different scenarios, running more than one offers additional sensitivity at the price of specificity and computation time. This is something we routinely perform to obtain better assemblies from a dataset (e.g., [28]).

Our results showed that identifying novel viruses (blastx vs. GenBank) is nearly as sensitive as identifying known viruses (blastn vs. GenBank). This is promising for the use of HTS as a replacement for biological indexing as a phytosanitary diagnostic tool [5,7].

Running NCBI blastx against GenBank was too computationally intense to evaluate for this study. We looked at two tractable alternative approaches that performed equally well. The first involved using a small database of virus proteins to obtain presumptive positives. The much smaller list of presumptive positives is then screened against GenBank using NCBI blastx. The second method uses DIAMOND blastx, a faster implementation of the NCBI translated search algorithm.

### 4.3. k-mer Database Approaches

The kraken2 k-mer method was a surprising standout, though it requires extensive computational and memory resources. While the method relies on exact substring matches, it can incorporate the genetic diversity of a virus by using a large target database (in this case, all of GenBank). The k-mer method was sensitive and also more specific than read mapping. We routinely filter spurious low-complexity matches to viral sequences when employing the read mapping approach. We also routinely filter misidentified and artifactual GenBank sequences (e.g., a series of host sequences amplified using degenerate primers and wrongly annotated as citrus exocortis viroids, as described in [29]). These types of sequences may be precluded from being assigned to a specific virus in the k-mer database due to their taxonomic ambiguity. What the method lacks is a consensus sequence that would allow for a better follow up, but this could be added by subsequent mapping or de novo assembly of the classified reads.

## 5. Recommendations

*An ensemble is best.* In general bioinformatics, tools are implemented as heuristic methods. That is, they are different ad hoc approaches used to solve a problem where the well-defined optimal solution may be intractable or may not exist. Each heuristic implementation gives slightly different results. In our diagnostic setting, we can exploit these implementation differences by combining assembly methods to achieve greater sensitivity. Translated annotation is required for sensitive detection of novel viruses; however, it is not a replacement for nucleotide methods that can detect untranslated homologies and are efficient enough to use on individual reads with potentially greater sensitivity. In this study, this recommendation is exemplified by the performance of the two ensemble diagnostics evaluated, which both yielded a higher level of diagnostic sensitivity than all individual methods.

*The more computational work you can put in, the greater the sensitivity.* This is not only a corollary of our first recommendation but also follows from the observation that the best annotation tools tend to be the most computationally expensive. For read mapping, the slower, more sensitive algorithm combined with a larger target database incorporating genetic diversity performed best. For de novo assembly, multiple assemblers may be run for improved performance, and expensive translated searches offer the potential to sensitively detect novel viruses. The k-mer method was the most sensitive technique for known viruses evaluated but it requires the largest computer to run.

*Curation improves diagnostic accuracy.* It is our experience at FPS that expert curation can be used to dramatically improve specificity over the numbers observed in this study. This is because these methods ascertain molecular sequences that can be further investigated by an expert. Curation can investigate homologies at the sequence and database level, ensuring the annotation is specific to the putative virus. Furthermore, it can be investigated at the literature level to determine the level of confidence and, likely, the host range of the virus homolog. Thus, specificity can be sacrificed somewhat for sensitivity. In our phytosanitary context, HTS is considered a presumptive test—largely due to the possibility of sample contamination. Presumptive positives will be followed up using PCR on an independent sample from the plant at a different time [7,8].

*The molecular protocol is a limiting step.* When positives are determined by a very small number of reads, it is important to use a sequencing protocol that is robust to cross contamination. For example, Illumina platforms can be prone to cross contamination within a multiplexed run by the misidentification of the attached barcode sequences and between runs via sample retention in the fluidics system [30]. We reduce this possibility using 96 dual index barcoding. Barcode pairs are more robust to misidentification within a run and allow for more infrequent re-use between runs. In the Illumina Nextseq, we employ additional washing steps to reduce carryover between runs. Finally, we note that a good ribo-depletion step is required to remove a large amount of unwanted material that would otherwise be sequenced. We note that our recommended sequencing depth is dependent on how efficient ribo-depletion is.

*For our specific ribo-depleted grapevine HTS protocol we recommend:* (1) Sequence to at least 15 million reads. The limit of detection analyses suggests diminishing returns after that point. (2) Host screening of the data upfront leads to improved sensitivity for de novo assembly. Since a majority of the reads are removed during this step, different hosts and ribo-depletion protocols may affect performance. (3) If employing a single de novo assembler, use spades followed by blast against the GenBank non-redundant nucleotide and protein databases. (4) For maximum sensitivity, employ the most sensitive approach available for mapping individual reads against a database that incorporates the known genetic diversity. In our study, this was bowtie2 using the very-sensitive local mode against the complete viral division of GenBank. Summing reads over taxa using a minimum depth cutoff of 20 and a minimum coverage cutoff of 200 bp removed the vast majority of false positives. We reduce those cutoffs when employed as a confirmatory test. (5) For highest sensitivity, use a read mapping method, de novo assembly and a k-mer method as a third check and a confirmation. In our study, 35 reads would be appropriate as a standalone test using kraken2 and could be reduced as a confirmatory test.

In conclusion, HTS has become a critical component of the management of grapevine viral diseases. It is distinct from previous widely deployed methods in that it benefits from but does not rely on previous characterization of the viral agent. This allows for sensitive detection of known viruses with the possibility of detecting novel viruses. In this paper, we empirically evaluated different bioinformatic tools for detecting viruses in HTS and reported our recommendations on its implementation in a phytosanitary setting. At FPS, we have implemented the ensemble method of de novo and read mapping (Section 3.6) since 2016. Because the diagnostic is inherently presumptive, we focus primarily on maximizing sensitivity. Because the protocol directly determines molecular sequences for a putative viral agent, it lends itself well to additional expert evaluation and follow up experimentation.

## Figures and Tables

**Table 1:** Composition and number of independent occurrences of virus and viroid infections in our grapevine validation panel. For this study, we considered PCR-confirmed infections to be “real” positives. N = number of panel samples.

Virus	Acronym	N	Virus	Acronym	N
Arabis mosaic virus	ArMV	2	Grapevine roditis leaf discoloration-assoc. virus	GRLDaV	1
Grapevine asteroid mosaic assoc. virus	GAMaV	3	Grapevine rupestris stem pitting-associated virus	GRSPaV	15
Grapevine badnavirus 1	GBV-1	1	Grapevine rupestris vein feathering virus	GRVFV	7
Grapevine enamovirus 1	GEV-1	1	Grapevine virus A	GVA	6
Grapevine fanleaf virus	GFLV	1	Grapevine virus B	GVB	5
Grapevine fleck virus	GFkV	2	Grapevine virus D	GVD	1
Grapevine Kizil Sapak virus	GKSV	1	Grapevine virus E	GVE	1
Grapevine leafroll-associated virus 1	GLRaV-1	3	Grapevine virus F	GVF	4
Grapevine leafroll-associated virus 2	GLRaV-2	4	Grapevine virus L	GVL	1
Grapevine leafroll-associated virus 3	GLRaV-3	9	Grapevine satellite virus	satGVV	1
Grapevine leafroll-associated virus 4	GLRaV-4	8	**Viroids**	**Acronym**	**N**
Grapevine leafroll-associated virus 7	GLRaV-7	1	Hop stunt viroid	HSVd	19
Grapevine polerovirus 1	GPoV-1	1	Grapevine yellow speckle viroid 1	GYSVd-1	17
Grapevine red blotch virus	GRBV	1	Grapevine yellow speckle viroid 2	GYSVd-2	9
Grapevine Red Globe Virus	GRGV	1	Australian grapevine viroid	AGVd	5

**Table 2:** Sample, sequencing and filtering data.

**Independent Grapevines**	19	
**Independent Samples**	38	
Average number of reads sequenced	24	million average
Total number of reads sequenced	903	million total
Average number of bp sequenced	1.77	billion bp average
Total number of bp sequenced	67.2	billion bp total
**In silico Subsampled Replicates**		
Number of subsamples	32	per sample
	1216	total
Number of reads sampled	12.4	billion total
Number of bp sampled	924.6	billion total
**Adapter and Quality Trimmed**		
Number of trimmed reads	12.4	billion total
	0.02%	% reduction
	918.2	billion total
	0.70%	% reduction
**Host Filtered**		
Number of reads post filter	4.0	billion total
	67.73%	% reduction
Number of bp post filter	296.4	billion total
	67.72%	% reduction

**Table 3:** Summary of diagnostic statistics for three popular read mapping algorithms using three databases. Both host-screened and unscreened reads were considered. Statistics are averaged over all in silico replicates sampled to a depth of at least 15 million reads. The observed trends are consistent with all in silico replicates.

Algorithm	Database	Host Screened	True-Positive Rate	False-Positive Rate	False-Negative Rate	True-Negative Rate
bowtie2	Ref-GV	yes	91.49%	0.55%	8.51%	99.45%
bowtie2-vsl			92.25%	0.54%	7.75%	99.46%
pathoscope2			92.25%	0.48%	7.75%	99.52%
bowtie2	NT-GV		94.11%	2.41%	5.89%	97.59%
bowtie2-vsl			94.18%	2.39%	5.82%	97.61%
pathoscope2			88.53%	2.25%	11.47%	97.75%
bowtie2	NT-Viral		95.39%	1.05%	4.61%	98.95%
bowtie2-vsl			95.97%	1.04%	4.03%	98.96%
pathoscope2			92.22%	1.00%	7.78%	99.00%
bowtie2	Ref-GV	no	91.63%	0.58%	8.37%	99.42%
bowtie2-vsl			92.15%	0.55%	7.85%	99.45%
pathoscope2			92.15%	0.48%	7.85%	99.52%
bowtie2	NT-GV		94.46%	2.53%	5.54%	97.47%
bowtie2-vsl			94.28%	2.72%	5.72%	97.28%
pathoscope2			89.15%	2.45%	10.85%	97.55%
bowtie2	NT-Viral		95.66%	1.25%	4.34%	98.75%
bowtie2-vsl			96.42%	1.21%	3.58%	98.79%
pathoscope2			92.18%	1.11%	7.82%	98.89%

**Table 4:** Summary of diagnostic statistics for different BLAST annotation algorithms with their appropriate GenBank non-redundant databases. The diagnostic statistics are averaged over all in silico replicates sampled at a depth of at least 15 million reads.

Algorithm	Detecting	True-Positive Rate	False-Positive Rate	False-Negative Rate	True-Negative Rate
blastn nt	Viruses + Viroids	92.18%	0.25%	7.82%	99.75%
blastx GV + NR	Viruses + Viroids	57.16%	0.24%	42.84%	99.76%
diamond NR	Viruses + Viroids	57.16%	0.24%	42.84%	99.76%
blastn NT	Viruses	91.98%	0.32%	8.02%	99.68%
blastx GV + NR	Viruses	91.92%	0.31%	8.08%	99.69%
diamond NR	Viruses	91.92%	0.31%	8.08%	99.69%
blastn NT	Viroids	91.36%	1.92%	8.64%	98.08%

## Data Availability

Sequence data available from the authors on request.

## Supplementary Materials

The following supporting information can be downloaded at: https://www.mdpi.com/article/10.3390/v16121957/s1, Table S1. Phytosanitary status of the 19 grapevine validation panel; Table S2. Table of Grapevine Viruses used in this study, their taxonomic identifiers, and selected reference sequences; Figure S1. Representative LOD curves for read mapping algorithms; Figure S2. Representative histograms for read mapping algorithms.
