# Supplementary material for: High-Throughput Sequencing for the Detection of Viruses in Grapevine: Performance Analysis and Best Practices
Source: Viruses. 2024 Dec 20;16(12):1957. doi: 10.3390/v16121957 (PMC11680390; doi:10.3390/v16121957)

Table S1. Phytosanitary status of the 19 grapevine validation panel.

Plant ID	Sample ID (Cane)	Sample ID (Petiole)	Phytosanitary Status
83824	H2493	H2518	GEV-1,GLRaV2,GLRaV3,GLRaV4,GRSPaV,GYSVd-1,HSVd
83846	H2494	H2520	GLRaV1,GLRaV3,GRSPaV,GVA,GVB,GVL,GYSVd-1,GYSVd-2,HSVd,GV-Sat
83852	H2495	H2519	GLRaV3,GLRaV4,GRSPaV,GRVFV,GYSVd-1,HSVd
83873	H2496	H2512	AGVd,GLRaV4,GRSPaV,GRVFV,GYSVd-1,GYSVd-2,HSVd
83886	H2497	H2513	AGVd,GLRaV3,GLRaV4,GRSPaV,GRVFV,GVF,GYSVd-1,GYSVd-2,HSVd
83916	H2498	H2525	AGVd,GBV1,GLRaV3,GLRaV4,GRLDaV,GRSPaV,GVA,GVB,GVF,GYSVd-1,GYSVd-2,HSVd
83948	H2499	H2526	GAMaV,GFLV,GLRaV3,GRVFV,HSVd
84010	H2500	H2527	AGVd,GKSV,GLRaV1,GLRaV7,GPoV-1,GRSPaV,GRVFV,GVA,GYSVd-1,GYSVd-2,HSVd
84014	H2501	H2528	AGVd,GLRaV3,GRSPaV,GRVFV,GVA,GYSVd-1,GYSVd-2,HSVd
84107	H2502	H2517	GRSPaV,GYSVd-1,HSVd
84112	H2503	H2531	GLRaV1,GLRaV3,GLRaV4,GRSPaV,GVA,GVF,GYSVd-1,HSVd
84124	H2504	H2524	GFkV,GLRaV2,GRBV,GRSPaV,GRVFV,GVB,GYSVd-1,HSVd
83940	H2505	H2529	GLRaV2,GLRaV4,GRSPaV,GRVFV,GVA,GVB,GVD,GVF,GYSVd-1,GYSVd-2,HSVd
84038	H2506	H2523	GLRaV3,GRSPaV,GVE,GYSVd-1,GYSVd-2,HSVd
84050	H2507	H2521	GFkV,GLRaV2,GRGV,GRSPaV,GVB,GYSVd-1,HSVd
84200	H2508	H2522	GLRaV4,GRSPaV,GYSVd-1,GYSVd-2,HSVd
87105	H2509	H2514	ArMV,GAMaV,GYSVd-1,HSVd
87104	H2510	H2515	ArMV,GAMaV,GYSVd-1,HSVd
50738	H2511	H2530	HSVd

Table S2. Table of Grapevine Viruses used in this study, their taxonomic identifiers, and selected reference sequences.

Species	Acronym	Part	TaxID	Positive
Potato virus X	PVX	Virus	12183	
Grapevine Kizil Sapak virus	GKSV	Virus	2650001	Y
Grapevine rupestris stem pitting-associated virus	GRSPaV	Virus	196400	Y
Grapevine virus T	GVT	Virus	2016035	
Grapevine berry inner necrosis virus	GINV	Virus	81877	
Grapevine Pinot gris virus	GPGV	Virus	1051792	
Grapevine virus A	GVA	Virus	35288	Y
Grapevine virus B	GVB	Virus	35289	Y
Grapevine virus D	GVD	Virus	51617	Y
Grapevine virus E	GVE	Virus	516956	Y

Grapevine virus F	GVF	Virus	1221437	Y
Grapevine virus K	GVK	Virus	2016034	Y
Grapevine virus G	GVG	Virus	2022475	
Grapevine virus H	GVH	Virus	2045345	
Grapevine virus I	GVI	Virus	2052157	
Grapevine virus J	GVJ	Virus	2093496	
Grapevine virus L	GVL	Virus	2283237	Y
Grapevine virus M	GVM	Virus	2571274	
Alfalfa mosaic virus	AMV	Virus	12321	
Cucumber mosaic virus	CMV	Virus	12305	
Grapevine angular mosaic virus	GaMoV	Virus	273001	
Grapevine virus S	GVS	Virus	1245593	
Grapevine line pattern virus	GLPV	Virus	2741672	
Tomato spotted wilt orthotospovirus	TSWV	Virus	1933298	
Grapevine vein clearing virus	GVCV	Virus	1050407	
Grapevine Roditis leaf discoloration-associated virus	GRLDaV	Virus	1471299	Y
Grapevine badnavirus 1	GBV1	Virus	2052838	Y
Grapevine leafroll-associated virus 1	GLRaV1	Virus	47985	Y
Grapevine leafroll-associated virus 3	GLRaV3	Virus	55951	Y
Grapevine leafroll-associated virus 4	GLRaV4	Virus	70177	Y
Grapevine leafroll-associated virus 13	GLRaV13	Virus	1815581	
Grapevine rootstock stem lesion associated virus	GLRaV2	Virus	167634	Y
Grapevine leafroll-associated virus 2	GLRaV2	Virus	64003	Y
Grapevine leafroll-associated virus 5	GLRaV4	Virus	71032	Y
Grapevine leafroll-associated virus 6	GLRaV4	Virus	203168	Y
Grapevine leafroll-associated virus 4 (Alternative Tax ID)	GLRaV4	Virus	367121	Y
Grapevine leafroll-associated virus 11	GLRaV4	Virus	446568	Y
Grapevine leafroll-associated virus 4 (Alternative Tax ID)	GLRaV4	Virus	446568	Y
Grapevine leafroll-associated virus 10	GLRaV4	Virus	367121	Y
Grapevine leafroll-associated virus Carn	GLRaV4	Virus	659661	Y
Grapevine leafroll-associated virus 4 (Alternative Tax ID)	GLRaV4	Virus	184610	Y
Grapevine Leafroll Associated Virus 9	GLRaV4	Virus	184610	Y
Grapevine endophyte endornavirus	GEEV	Virus	1249676	
Grapevine begomovirus A	GBVA	Virus	2793638	
Grapevine red blotch virus	GRBV	Virus	1381007	Y
Wild Vitis latent virus 1	WVV1	Virus	2560839	
Temperate fruit-decay-associated virus	TFDaV	Virus	1628899	
Grapevine geminivirus A	GGVA	Virus	1906317	
Grapevine leafroll-associated virus 7	GLRaV7	Virus	217615	Y

Grapevine enamovirus 1 (Alternative Tax ID)	GEV-1	Virus	1892570	Y
Grapevine enamovirus 1	GEV-1	Virus	2560515	Y
Grapevine cryptic virus	GCV	Virus	1256788	
Grapevine Garan dmak virus	GGDV	Virus	2601258	
Grapevine Muscat rose virus	GMRV	Virus	2601259	
Bean common mosaic virus	BCMV	Virus	12196	
Potato virus Y	PVY	Virus	12216	
Grapevine Cabernet Sauvignon reovirus	GCSV	Virus	1640277	
Apple latent spherical virus	ALSV	Virus	101688	
Broad bean wilt virus	BBMV	Virus	95622	
Grapevine fabavirus	GFabV	Virus	1849838	
Arabis mosaic virus	ArMV	Virus	12271	Y
Grapevine chrome mosaic virus	GCMV	Virus	12273	
Grapevine fanleaf virus	GFLV	Virus	12274	Y
Tomato black ring virus	TBRV	Virus	12275	
Tomato ringspot virus	ToRSV	Virus	12280	
Tobacco ringspot virus	TRSV	Virus	12282	
Cherry leafroll virus	CLRV	Virus	12615	
Raspberry ringspot virus	RpRSV	Virus	12809	
Blueberry leaf mottle virus	BBLMV	Virus	38172	
Artichoke Italian latent virus	AILV	Virus	46075	
Peach rosette mosaic virus	PRSM	Virus	65068	
Grapevine Anatolian ringspot virus	GARSV	Virus	223769	
Grapevine deformation virus	GDeV	Virus	233784	
Grapevine Bulgarian latent virus	GBLV	Virus	748667	
Grapevine Tunisian ringspot virus	GTRV	Virus	2052649	
Strawberry latent ringspot virus	SLRSV	Virus	28351	
Grapevine polerovirus 1	GPoV-1	Virus	2696999	Y
Carnation mottle virus	CarMV	Virus	11986	
Tobacco necrosis virus D	TNV-D	Virus	12056	
Grapevine Algerian latent virus	GALV	Virus	208084	
Petunia asteroid mosaic virus	PAMV	Virus	270255	
Grapevine-associated tymo-like virus	GaTLV	Virus	2338396	
Grapevine fleck virus	GFkV	Virus	103722	Y
Grapevine redglobe virus	GRGV	Virus	103723	Y
Grapevine rupestris vein feathering virus	GRVFV	Virus	204933	Y
Grapevine Syrah virus 1	GSyV1	Virus	630199	
Blackberry virus S	BlVS	Virus	670883	
Grapevine asteroid mosaic-associated virus	GAMaV	Virus	2169998	Y

Raspberry bushy dwarf virus	RBDV	Virus	12451	
Sowbane mosaic virus	SoMV	Virus	378833	
Grapevine satellite virus	GV-Sat	Virus	1343493	Y
Tobacco mosaic virus	TMV	Virus	12242	
Tomato mosaic virus	ToMV	Virus	12253	
Grapevine virga-like virus	GVLV	Virus	2572116	
Hop stunt viroid	HSVd	Viroid	12893	Y
Grapevine yellow speckle viroid-1	GYSVd-1	Viroid	12904	Y
Grapevine yellow speckle viroid-2	GYSVd-2	Viroid	46342	Y
Australian grapevine viroid	AGVd	Viroid	190810	Y

Figure S1 Representative LOD curves for read mapping algorithms.

Representative LOD curves for the most sensitive and most specific mapping algorithms bowtie2vsl vs NT-Viral and Pathoscope vs Ref-GV. The curves have an asymptotic quality and at 15M reads obvious flattening has occurred. The slope at 15M reads was estimated using a +/- 1M window: dy/dx for pathoscope is 2.0×10^{-9} and bowtie2vsl is 1.9×10^{-9}, corresponding to an increase of 1% TPR for 5M per reads.

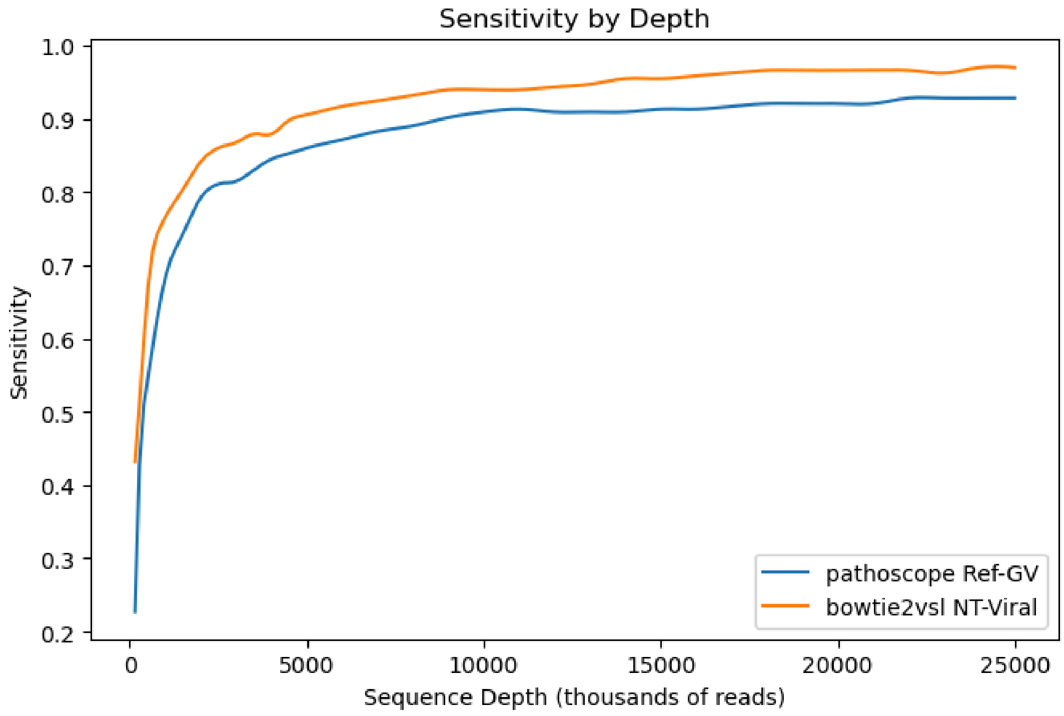

Figure S2. Representative histograms for read mapping algorithms.
Representative histograms for the most sensitive and most specific mapping algorithms. (a) bases covered for Pathoscope vs Ref-GV (b) bases covered for bowtie2vsl vs NT-Viral (c) mapped reads for Pathoscope vs Ref-GV (d) mapped reads for bowtie2vsl vs NT-Viral.

(a)

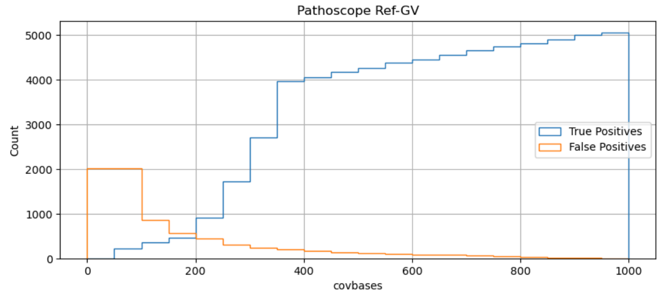

(b)

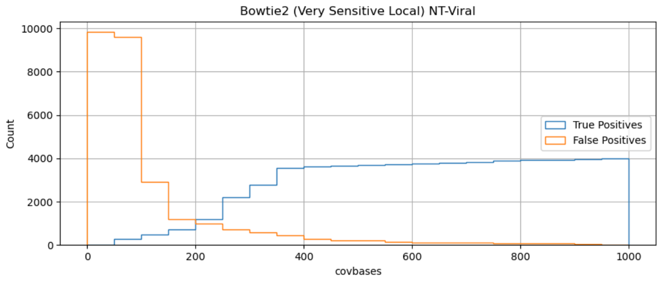

(c)

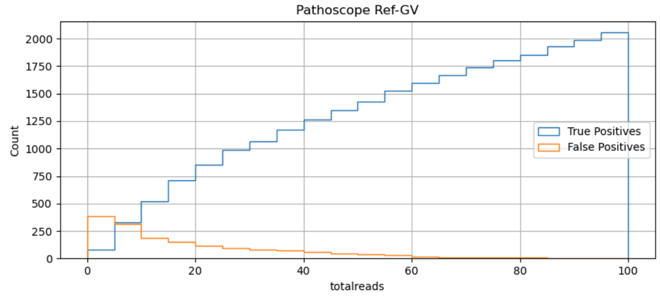

(d)

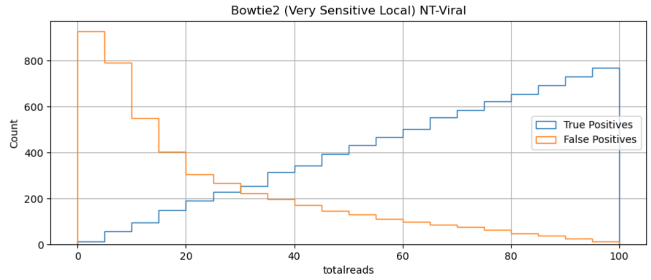

Supplement: Supplementary file 1 [file viruses-16-01957-s001.zip › viruses-3346352-supplementary.pdf]
